# Factors associated with psychological stress and distress among Korean adults: the results from Korea National Health and Nutrition Examination Survey

**DOI:** 10.1038/s41598-020-71789-y

**Published:** 2020-09-15

**Authors:** Yejin Cheon, Jinju Park, Bo Yoon Jeong, Eun Young Park, Jin-Kyoung Oh, E Hwa Yun, Min Kyung Lim

**Affiliations:** 1 Department of Cancer Control and Population Health, Graduate School of Cancer Science and Policy, National Cancer Center, 323 Ilsan-ro, Ilsandong-gu, Goyang-si, Gyeounggi-do 10408 Republic of Korea; 2 Division of Cancer Prevention and Early Detection, National Cancer Control Institute, National Cancer Center, 323 Ilsan-ro, Ilsandong-gu, Goyang-si, Gyeounggi-do 10408 Republic of Korea

**Keywords:** Health care, Risk factors

## Abstract

The prevalence of stress and distress has been increasing and being important public health issues; nevertheless, few studies have assessed the factors associated at the population level. This study identified factors associated and how they differentially influence stress and distress. A total of 35,105 individuals aged 19 years and older using nationally representative data from the Korea National Health and Nutrition Examination Survey (2007–2012) were included in the study. Subjects were differentiated by gender and psychological state (no symptoms, stress, distress). The associations of socio-demographics, psychosocial factors, health behaviours, and chronic illness with psychological states were analysed by gender. Socio-demographics and psychosocial factors such as lower household income, lower education level, living alone or negative outcome of marriage, and unemployment were associated with distress in both genders. Male and female educated higher and with short sleep duration, male living alone and with higher household income, and female married and with a lower household income was associated with stress. A perceived body image of slim or fat was associated with distress and stress in both genders. Behavioural factors, such as smoking, higher alcohol consumption, and abnormal calorie intake, were associated with stress and distress in both genders, with the exception of alcohol consumption in distress and abnormal calorie intake in stress of male. Socio-economic deprivation and negative psychosocial and behavioural factors were differently associated with psychological distress or stress by gender. Intervention strategies for distress and stress should be specifically tailored regarding these differences.

## Introduction

Psychological health is unquestionably an important issue, given that people with psychological symptoms accounts large proportion of the population worldwide. 75 percent of Americans report experiencing at least one symptom of stress in the past month and around one-fourth American said stress has a strong impact on their physical or mental health^1^. Four out of 10 UK adults feel stressed most days, and one-third Australians experienced depressive symptoms^2^. Koreans who feel their life is substantially or extremely stressful accounted for 27.9% and the prevalence of depression was 5.6% in the same population^3^. Consistent with these trends, the terms ‘psychological stress’ and ‘psychological distress’, which are defined as having perceived pressure from daily life but coping with it and stress exceeding persons’ ability to manage it, respectively, are increasingly used in social, academic, and employment settings^4,5^.

Several factors have been investigated with respect to their role in psychological stress and distress: socio-demographics, health behaviours, and the psychosocial environment. Possible health behaviours associated with psychological stress and distress include tobacco use, alcohol consumption, physical inactivity, dietary intake, and weight change^6–8^. Psychosocial environmental factors that have been investigated include marital status, living alone, employment status, shift work, working hours, weight change, body mass index (BMI), perceived body image (PBI), and skipping meals^7,9–16^. Gender, age, household income, education, and occupation are sociodemographic factors that have been investigated^6,7^. Clinical illness such as pulmonary disease, asthma, hypertension, diabetes mellitus, dementia, mental disability, cancer, and degenerative diseases, has been also studied as a strong predictors of psychological stress and distress in some of previous studies focusing on the association of a specific chronic illness^17^.

However, the associations found between the aforementioned factors and psychological stress and distress have been inconsistent due to differences in study design, target population, and other potential factors included in analyses, and there are lack of studies to include potential factors associated with psychological stress and distress, comprehensively.

Therefore, an exploratory study, which comprehensively investigate sociodemographic, behavioural, psychosocial, and chronic illness factors associated with psychological stress and distress, and examine how those factors may differentially influence stress and distress, has been done using nationally representative population-based data from Korea.

## Data and methods

### Study design and participants

The study design was cross-sectional, using data from Korea National Health and Nutrition Examination Survey (KNHANES), which is a national survey designed and conducted by the Ministry of Health and Welfare and the Korean Centers for Disease Control and Prevention (https://knhanes.cdc.go.kr/knhanes/index.do) with the national representative sample through the multi-stage probability sampling method and structured and validated questionnaire. Korean Centers for Disease Control and Prevention’s Institutional Review Board approved the protocols of the KNHANES. In this study, we used a dataset of the KNHANES that is open to the public. The Fourth (2007–2009) and Fifth (2010–2012) KNHANES included 50,405 participants. For the current study, subjects were excluded who were under 19 years of age, or who had incomplete information on psychological state. The final study population comprised 35,105 subjects—14,879 males and 20,226 females.

### Data and measurements

Psychological state was defined by the answers to three questions: Q1, ‘How much do you feel stress in your daily life?’ (response to the question is ‘minimally stressful’ or ‘moderately stressful’ or ‘substantially stressful’ or ‘extremely stressful’); Q2, ‘Have you ever experienced suicidal ideation within the last year?’ (response to the question is ‘yes’ or ‘no’); and Q3, ‘Have you ever suffered from feeling down, depressed, or hopeless for two consecutive weeks or longer during the last year?’ (response to the question is ‘yes’ or ‘no’).

Subjects who responded to question 1 that their lives were ‘substantially stressful’ or ‘extremely stressful’ but responded ‘no’ to both question 2 and 3 were categorized as experiencing psychological stress alone, which means having more than substantial level of pressure from daily life but coping with it. Without regard to if they have psychological stress or not, subjects who concurred with questions 2 or 3 were categorized as experiencing psychological distress, which could be defined as having stress exceeding persons’ ability to manage it. Subjects who did not report any daily stress, melancholy, or suicidal ideation were categorized as the no symptom group.

Data obtained from the survey questionnaire, which has been validated by Korean CDC for National Survey, and anthropometric measurements included the following: sociodemographic factors such as gender (male, female), age (19–29, 30–39, 40–49, 50 years or older), residence area (country, city, metropolitan city), education level (middle school or less, high school, college or more), and household income (lowest quartile, second quartile, third quartile, highest quartile); psychosocial factors such as marital status (single; married; bereaved, divorced, or separated), living alone without family member (yes, no), employment status (employed, employed, retired, students, and subjects who do not need an employment; unemployed, unemployed, between jobs, taking leave of absence), PBI (slim, normal, fat), BMI (low weight, < 18.5 kg/m^2^; normal, ≥ 18.5 kg/m^2^ and < 25 kg/m^2^; obese: ≥ 25 kg/m^2^)^18^, and sleep duration (short, less than 6 h per day; optimal, 6–8 h per day; long, more than 8 h per day); behavioural factors such as smoking status (never a smoker, former smoker, current smoker), amount of alcohol consumption per occasion (0 g, 0–24 g, 24–72 g, over 72 g of absolute alcohol concentration), physical activity (active, engaging in moderate or vigorous physical activity; inactive), and calorie intake (low, consuming lower than their basal metabolic rate (BMR); normal, consuming over their BMR and lower than 1.2 times their BMR; excessive, consuming more than 1.2 times their BMR)^19,20^; and physiological condition such as menstruation (yes, no) and clinical illness (yes, having illness or diseases such as (hypertension, dyslipidemia, stroke, myocardial infarction/angina pectoris, degenerative arthritis, rheumatoid arthritis, tuberculosis, asthma, diabetes mellitus, thyroid disease, atopic dermatitis, renal failure, hepatitis B and C, liver cirrhosis, chronic obstructive pulmonary disease, cancer, and degenerative diseases; no).

The following variables were considered in the initial analysis to regard predictors of psychological stress and distress, comprehensively, based on previous knowledge, but later excluded due to no significant association in the univariate analysis or a significant correlation with other variables included in the final analysis: occupation, frequency of alcohol consumption, diagnosis of depression, EQ-5D index (mobility, self-care, usual activities, pain/discomfort and anxiety/depression), distorted body image, trial to manage weight, weight change within a year, number of meals with their family in a day, frequency of dining out, skipping meals for last 2 days, working hours, and shift work.

### Statistical analysis

Chi-square tests were used to analyse frequencies of the distribution of baseline characteristics of the study participants and the effect size on the difference of the frequency distribution in each variable is measured. Prevalence ratio and associations between factors and psychological states including stress and distress were evaluated using multivariate logistic regression analysis adjusted with age only and all variables as appropriate^21^. p-for trend test has been done to identify the dose response relationship. All statistical tests were two-tailed with a 5% level of significance, and obtained using SAS version 9.3.

### Declaration of Helsinki

All methods were carried out in accordance with relevant guidelines and regulations.

### Ethics approval

All KNHANES surveys were conducted with informed consent of participants by Korea Centers for Disease Control and Prevention (KCDC) and the Institutional Review Board of KCDC approved the protocols of the KNHANES. In this study, we used a dataset of the KNHANES that is open to the public for retrospective analysis did not include personally identifiable information.

## Results

### Frequency distribution of potential factors by psychological status

Compared with the no symptom and psychological stress alone groups, both male and female subjects with psychological distress were more likely to be older, live in country, have lower education level, have lower household income, have negative marriage outcome (bereaved, divorced, or separated), live alone without family members, be unemployed, have a slim PBI, have shorter or longer sleep duration, have ever been smokers, be non-drinkers, have a low calorie intake, and have chronic illness. The distribution of these factors was inversed among subjects with psychological stress alone when they were compared with the no symptom group. Male subjects with psychological distress also tended to have low BMI, while female subjects with psychological distress tended to have high BMI, to be physically active, and to be undergoing menopause (Table 1).

**Table 1:** Difference on distribution of sociodemographic, psychosocial, and behavioral factors in study subjects by gender and psychological status.

Variables	Male										Female									
	Total		No symptom		Psychological stress		P-value^a^	Psychological distress		P-value^b^	Total		No symptom		Psychological stress		P-value^a^	Psychological distress		P-value^b^
	n = 14,879	%	n = 10,089	%	n = 2,379	%		n = 2,411	%		n = 20,226	%	n = 11,628	%	n = 2,880	%		n = 5,718	%	
**Age (years)**							< 0.0001			< 0.0001							< 0.0001			< 0.0001
19–29	1,840	12.9	1,264	12.5	346	14.5		230	9.5		2,526	11.4	1,312	11.3	548	19.0		666	11.6	
30–39	2,800	19.6	1,709	16.9	730	30.7		361	15.0		3,984	18.8	2,409	20.7	731	25.4		844	14.8	
40–49	2,852	19.7	1,849	18.3	608	25.6		395	16.4		3,708	18.3	2,305	19.8	526	18.3		877	15.3	
≥ 50	7,387	47.8	5,267	52.2	695	29.2		1,425	59.1		10,008	51.5	5,602	48.2	1,075	37.3		3,331	58.3	
**Region**							< 0.0001			< 0.0001							0.1303			< 0.0001
Metropolitan city	6,585	45.0	4,478	44.4	1,133	47.6		974	40.4		9,091	44.8	5,320	45.8	1,316	45.7		2,455	42.9	
Urban	5,017	33.8	3,346	33.2	870	36.6		801	33.2		6,839	33.6	3,924	33.7	1,016	35.3		1,899	33.2	
Rural	3,277	21.2	2,265	22.5	376	15.8		636	26.4		4,296	21.6	2,384	20.5	548	19.0		1,364	23.9	
**Education level**							< 0.0001			< 0.0001							< 0.0001			< 0.0001
Middle school or less	4,619	28.5	3,128	31.1	416	17.5		1,075	44.8		8,836	45.6	4,728	40.7	938	32.6		3,170	55.6	
High school	5,376	37.0	3,730	37.1	873	36.8		773	32.2		6,466	31.7	3,943	34.0	975	33.9		1,548	27.1	
College or more	4,843	34.5	3,208	31.9	1,085	45.7		550	22.9		4,885	22.7	2,939	25.3	961	33.4		985	17.3	
**Household income (Quartile)**							< 0.0001			< 0.0001							0.1544			< 0.0001
Lowest	2,721	16.6	1,782	18.0	252	10.8		687	29.1		4,330	22.4	2,078	18.2	512	18.1		1,740	31.1	
Second	3,716	25.0	2,523	25.4	548	23.4		645	27.3		5,035	25.2	2,833	24.8	748	26.5		1,454	26.0	
Third	4,063	28.8	2,805	28.2	731	31.2		527	22.3		5,228	26.4	3,191	27.9	738	26.1		1,299	23.2	
Highest	4,129	29.6	2,816	28.4	813	34.7		500	21.2		5,260	26.0	3,332	29.1	828	29.3		1,100	19.7	
**Marital status**							< 0.0001			< 0.0001							< .0001			< 0.0001
Single	2,411	15.9	1,600	15.9	423	17.9		388	16.1		2,369	10.8	1,235	10.6	498	17.3		636	11.2	
Married	11,640	79.4	7,984	79.4	1,878	79.3		1,778	74.0		13,820	68.3	8,324	71.8	2,012	70.0		3,484	61.1	
Bereaved/divorced/separated	774	4.7	468	4.7	68	2.9		238	9.9		3,985	20.9	2,038	17.6	366	12.7		1,581	27.7	
**Living alone without family members**							0.0031			< 0.0001							0.0014			< 0.0001
Yes	633	3.6	390	3.9	62	2.6		181	7.5		1795	9.4	897	7.7	172	6.0		726	12.7	
No	14,241	96.4	9,697	96.1	2,316	97.4		2,228	92.5		18,431	90.6	10,731	92.3	2,708	94.0		4,992	87.3	
**Employment status**							< 0.0001			< 0.0001							0.105			< 0.0001
Employed	12,834	88.8	8,829	87.9	2,189	92.4		1,816	76.0		12,723	62.7	7,588	65.7	1,925	67.3		3,210	56.7	
Unemployed	1967	11.2	1,214	12.1	181	7.6		572	24.0		7,361	37.3	3,969	34.3	937	32.7		2,455	43.3	
**PBI**							< 0.0001			< 0.0001										< 0.0001
Slim	3,265	20.9	2,100	20.8	503	21.2		662	27.5		2,984	14.7	1,504	12.9	428	14.9	< .0001	1,052	18.4	
Normal	6,160	41.9	4,413	43.7	810	34.1		937	38.9		8,148	40.8	4,956	42.6	1,077	37.4		2,115	37.0	
Fat	5,450	37.2	3,574	35.4	1,065	44.8		811	33.7		9,093	44.5	5,167	44.4	1,375	47.7		2,551	44.6	
**BMI**							0.0001			0.0002							< .0001			< 0.0001
Low weight	487	3.0	317	3.2	59	2.5		111	4.6		1,109	5.3	581	5.1	211	7.4		317	5.6	
Normal	9,136	61.5	6,238	62.2	1,381	58.4		1,517	63.4		12,955	64.9	7,548	66.0	1,862	65.4		3,545	62.7	
Obesity	5,166	35.5	3,476	34.6	926	39.1		764	31.9		5,878	29.8	3,307	28.9	776	27.2		1,795	31.7	
**Sleep duration**							0.0001			< 0.0001							< .0001			
Short	2,019	12.3	1,214	12.0	325	13.7		480	19.9		3,601	17.6	1,679	14.4	551	19.1		1,371	24.0	< 0.0001
Optimal	11,747	80.7	8,131	80.6	1,931	81.2		1,685	69.9		14,895	73.7	8,982	77.2	2,117	73.5		3,796	66.4	
Long	1,113	7.0	744	7.4	123	5.2		246	10.2		1,730	8.8	967	8.3	212	7.4		551	9.6	
**Smoking status**							< 0.0001			< 0.0001							< 0.0001			< 0.0001
Never	3,217	22.2	2,295	22.8	476	20.0		446	18.5		18,303	90.7	10,807	93.0	2,571	89.3		4,925	86.2	
Former	5,393	36.6	3,872	38.4	689	29.0		832	34.5		799	3.9	373	3.2	130	4.5		296	5.2	
Current	6,262	41.2	3,918	38.8	1,212	51.0		1,132	47.0		1,114	5.4	444	3.8	177	6.2		493	8.6	
**Alcohol consumption**							< 0.0001			0.0126							< 0.0001			< 0.0001
0 g	2,523	16.4	1,796	17.8	243	10.2		484	20.1		7,760	39.5	4,450	38.3	917	31.8		2,393	41.9	
0–24 g	2,277	15.3	1,616	16.0	288	12.1		373	15.5		6,868	33.9	4,168	35.8	998	34.7		1,702	29.8	
24–72 g	4,915	33.5	3,389	33.6	782	32.9		744	30.9		4,354	20.9	2,459	21.2	724	25.1		1,171	20.5	
> 72 g	5,155	34.9	3,283	32.6	1,064	44.8		808	33.5		1,237	5.7	546	4.7	241	8.4		450	7.9	
**Physical activity**							0.2722			0.0022										0.0015
Inactive	11,208	74.9	7,533	74.7	1,802	75.8		1,873	77.7		16,217	80.3	9,412	81.0	2,295	79.7	0.1350	4,510	78.9	
Active	3,660	25.1	2,548	25.3	575	24.2		537	22.3		3,999	19.7	2,212	19.0	583	20.3		1,204	21.1	
**Calorie intake**							0.3373			0.0152							0.0024			< 0.0001
Low	2,065	16.3	1,381	16.2	309	16.9		375	18.8		4,918	26.4	2,618	24.3	710	27.3		1,590	30.6	
Normal	1,896	15.5	1,342	15.7	264	14.4		290	14.5		3,338	18.2	2,002	18.6	435	16.8		901	17.3	
Excess	8,385	68.1	5,798	68.0	1,254	68.6		1,333	66.7		10,301	55.4	6,142	57.1	1,452	55.9		2,707	52.1	
**Menstruation**																	< 0.0001			< 0.0001
Yes	–	–	–	–	–	–	–	–	–	–	9,256	46.0	5,485	49.1	1,631	59.8		2,140	39.5	
No	–	–	–	–	–	–		–	–		10,048	54.0	5,675	50.9	1,098	40.2		3,275	60.5	
**Chronic illness**							< 0.0001			< 0.0001							0.0003			< 0.0001
Yes	6,509	42.1	4,402	43.6	845	35.5		1,262	52.3		9,627	48.7	5,212	44.8	1,184	41.1		3,231	56.5	
No	8,370	57.9	5,687	56.4	1,534	64.5		1,149	47.7		10,599	51.3	6,416	55.2	1,696	58.9		2,487	43.5	

^a^Chi-square test for no symptom vs. psychological stress alone.

^b^Chi-square test for no symptom vs. psychological distress.

### The association of sociodemographic, psychological, and behavioural factors with psychological stress alone and distress

**Figure 1:**
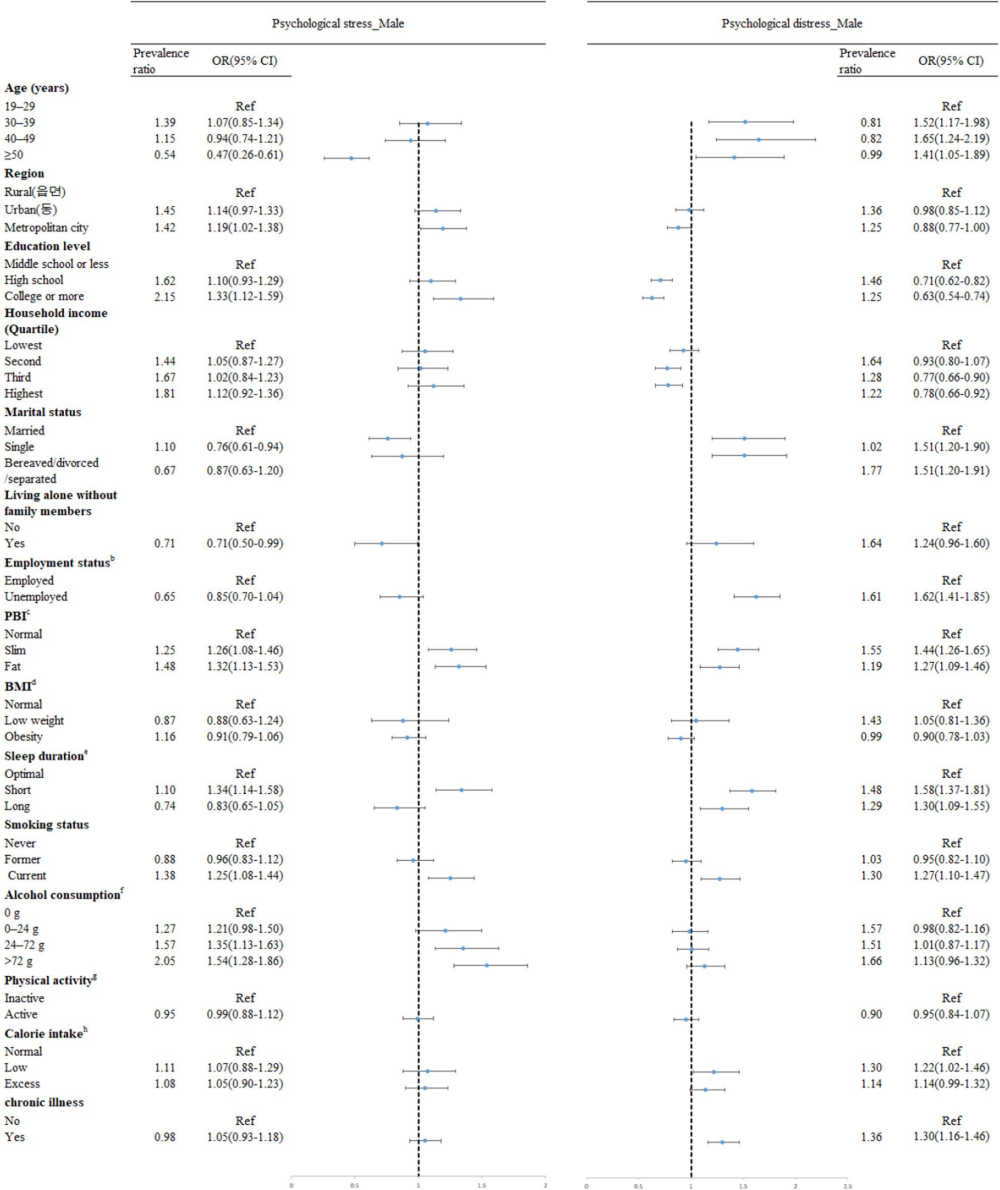
Forest plot showing odds ratio (OR) and 95% confidence interval (CI) for psychological stress and distress among male.

**Figure 2:**
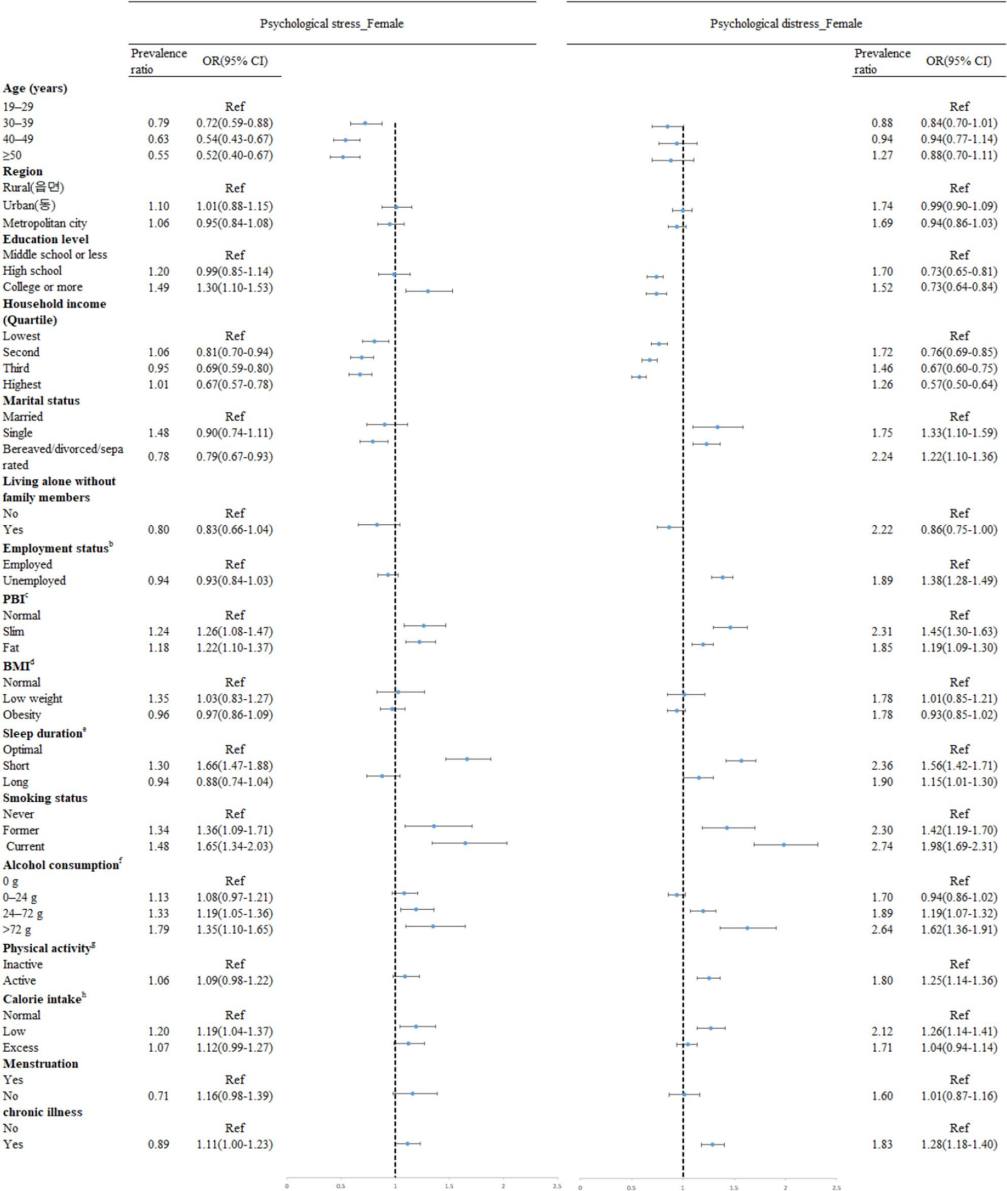
Forest plot showing odds ratio (OR) and 95% confidence interval (CI) for psychological stress and distress among female.

The current findings show the associations between various factors and psychological stress and psychological distress (Figs. 1, 2). After adjustment for all variable as appropriates, increased prevalence of psychological stress was shown in both gender being younger, more educated (odds ratio, OR 1.33, 95% confidence interval, CI 1.12–1.59 in male educated college or more; OR 1.30; 95% CI 1.10–1.53 female educated college or more), having slim or fat PBI (OR 1.32; 95% CI 1.13–1.53 in male with fat PBI; OR 1.22; 95% CI 1.10–1.37 in female with fat PBI), having short sleep duration (OR 1.34; 95% CI 1.14–1.58 in male, OR 1.66; 95% CI 1.47–1.88 in female), being current smokers (OR 1.25; 95% CI 1.08–1.44 in male, OR 1.65; 95% CI 1.34–2.03 in female), drinking higher amount of alcohol (OR 1.54; 95% CI 1.28–1.86 in male drinking more than 72 g/day; OR 1.35; 95% CI 1.10–1.65 in female drinking more than 72 g/day) Living in metropolitan city and living with family members were factors associated with increased prevalence of psychological stress in male only, while married, having a low calorie intake, and having chronic illness were in female only. Having higher household income decreased the odds of psychological stress among female.

Being different from the results in factors associated with psychological stress, for both males and females, relatively higher level of education (OR 0.63; 95% CI 0.54–0.74 in male educated college or more, OR 0.73; 95% CI 0.64–0.84 in female educated college or more) and household income (OR 0.78; 95% CI 0.66–0.92 in male at the highest income group, OR 0.57; 95% CI 0.50–0.64 in female at the highest income group) decreased the odds of psychological distress. Negative marriage outcome such as bereaved, divorced, and separated (OR 1.51; 95% CI 1.20–1.91 in male; OR 1.22; 95% CI 1.10–1.36 in female) and being single (OR 1.51; 95% CI 1.20–1.90 in male; OR 1.33; 95% CI 1.10–1.59 in female), unemployed (OR 1.62; 95% CI 1.41–1.85 in male; OR 1.38; 95% CI 1.28–1.49 in female), and having chronic illness (OR 1.30; 95% CI 1.16–1.46 in male; OR 1.28; 95% CI 1.18–1.40 in female) were associated with having psychological distress, even though these were not significantly associated with psychological stress. Having either slim or fat PBI, having either short or long sleep duration, being current smokers, having a low calorie intake were associated with increased prevalence of psychological distress, too, for both male and female. Having more amounts of alcohol consumption and being physically active increased the odds of psychological distress in female only.

## Discussion

In this study, we investigated the associations between various sociodemographic, psychosocial environment, and health behaviour factors and psychological stress and distress using nationally representative survey data (KNHANES). Different degrees and trends of associations were found between the different factors and psychological stress alone and distress. Additionally, these associations differed by gender.

More than substantial level of psychological stress coped with daily life was more prevalent among male or subjects aged in younger, while psychological distress which is severe mental problem such as feeling depressed or hopeless and suicidal ideation was more prevalent in female or subjects aged in older. The results correspond to previous studies reported that female are more psychologically and physiologically fragile for exposure to stressors and appeal more their negative emotion and mental symptom than male^22,23^.

Regarding the association of socio-demographic and psychosocial factors with the psychological distress among both male and female, a plausible explanation for these results is that most distress is caused by a loss of social support or relationships and worsening economic situations such as those caused by aging, living without a spouse, and being unemployed. The effects of those factors would be increased among those male aged in older, for whom retirement and bereavement are more frequent than among aged in younger. While, psychological stress might be initiated by conflict with people in their surroundings, when they live with other family members including their spouse or struggle to get better conditions such as higher income or promotion in position, and the effect has been furthered among subjects aged in younger and higher education level. Psychological stress was increased in higher household income among male, but it was inversed in female. Male are usually responsible for earning and could be in more stressful when they try to get more money, while female are usually responsible for spending and could be more stressful when they are in lack of money.

That the highest odds of distress were found for those with the lowest household income level is consistent with previous findings that financial difficulty increases psychological distress^14,24^. Correspondingly, a study showed that loss of employment is linked to increased distress (study was done in Norway among general population)^13^, as it may lead to financial strain^16,25^. Since males often have greater actual or perceived financial responsibility than females, this may lead to greater psychological impacts in men after retirement or loss of job^26^. Regarding the previous studies on educational differences in mental health problems, low education may be more consistently associated with severe mental health problems than minor psychiatric morbidity^23,27–30^, and it correspond with findings from current studies. Positive associations between being unmarried, bereaved, or divorced and psychological distress have been reported in previous studies, but there have been no such associations reported with psychological stress^16,24,25^. As shown in longitudinal studies including a study done for working-age population in Finland^7,14^, there was significant association between living alone and psychological distress in the current study.

Both slim and fat PBI showed significant associations with stress and distress among both males and females, while there was no significant association with BMI and either psychological state in either gender. This could indicate that perceived body image is a more important predictor of stress and distress than objectively measured bodyweight, even though many previous studies have suggested that unhealthy bodyweight, rather than perceived body image, predicts a higher prevalence of stress, depressive mood, and suicide attempts^10,11,15,31,32^. Interestingly, in the present study, slim PBI was also linked with higher odds of stress and distress than normal PBI in female. Furthermore, the odds of a slim PBI were relatively higher in the distressed group. As a study on the effect of distorted body weight perception on suicidal ideation among female in Korea suggested^33^, people who perceived their own bodies as abnormally slim usually had low body weight, but a large proportion of people who perceived their own bodies as abnormally fat were not objectively overweight. It is possible that slim PBI combined with low body weight is associated with distress or other specific environmental factors and unhealthy conditions that cause low body weight, thereby explaining the increased distress. Fat PBI without abnormally higher body weight could be linked more to stress than to distress.

We found that both short and long sleep duration was associated with increased distress in both gender, but only short sleep duration was associated with increased stress. Different study designs, target populations, and definitions of short and long sleep duration could cause inconsistent associations between sleep duration and psychological stress and distress^34–39^. Such U-shaped associations have also been found in a US study on postmenopausal women^40^, as well as some of previous studies that found associations of both insomnia and oversleeping with psychosocial stress and distress^35,36,38,39^.

Health behavioural factors such as current smoking, higher amount of alcohol drinking, and excessive calorie intake were associated with either psychological distress or stress among male. Among females, most health behaviours showed significant associations with both distress and stress (with the exception of physical activity and psychological stress), such as having ever been a smoker, being physically active, and either a lack of or excessive calorie intake. As suggested in previous studies, stress may increase (or decrease) appetite leading to greater (or lesser) calorie intake; additionally, changing body weight may be a cause of stress and distress^41,42^. Smoking and alcohol drinking are well-known stress behaviours and stress has been identified as a barrier to the uptake of behaviour change, although some studies suggest it may need to reach before impacting on behaviours^6, 7,43,44^. We found significant associations with stress and distress among females for both smoking and alcohol drinking, although only alcohol drinking was associated with stress among males. In Korea, as approximately 40% of males are current smokers and drinking alcohol is also more prevalent among males than in females^3^, it is difficult to identify differences among males regarding those behaviours.

Having chronic illness was associated with distress and stress in female, while the association was found in distress for men. Although some recent studies suggest psychological stress may increase the onset of chronic diseases, there are lots of room to be explained to confirm the findings because the role of psychological stress on disease developments and its’ interaction with disease-prone behaviours have not been distinguished, yet^17^.

These findings support the transactional model which emphasise environmental stimuli as stressors and social, psychological, and biological factors that affect both the occurrence of, and responses to, potential stressors^5,45^. Socio-demographic factors and psychosocial factors of the current study might be considered environmental stressors, and health behaviours and chronic illness could be explained as factors associated with occurrence of stress or results of response to stress. Furthermore, the current study added evidence on the association of various potential factors on psychological stress and distress with the representative sample of general population, which are in lack and need further study more.

Although the current study provided an overview of the factors associated with stress and distress among the general population in Korea using data from a nationwide population-based survey, it nonetheless had several limitations. First, the cross-sectional design means that temporality cannot be evaluated. Second, although self-reported data are commonly used in observational studies, such data are prone to recall bias and misclassification. Third, measures of the psychological state were obtained using only three questions, potentially resulting in imprecise data and misclassification of psychological states. However, these questions have been used in the KNHANES since 1998 as health indicators representing mental health with expert consensus, and previous study results obtained from these questions have concurred with results from other data sources. Fourth, stressful life events were not comprehensively assessed, although some such events were considered, such as living alone, negative outcomes of marriage, unemployment, overworking, and shift work.

Consequently, the current study found that sociodemographic factors, psychosocial factors, and health behaviours have different associations with psychological states with regard to their direction of association, type of stress (defined as stress and distress), and gender. However, psychosocial factors were suggested as important with respect to distress in both males and females, as well as stress among females in Korea. Therefore, specific approaches tailored to both the target population and psychological state are required to prevent and control psychological stress alone and distress. In addition, based on these results, further studies are warranted to help gain a better understanding of factors associated with psychological states.
